# Through Doorways and Down Corridors: Investigating Asymmetries During Computer Maze Navigation

**DOI:** 10.5334/joc.92

**Published:** 2020-02-03

**Authors:** Nicole A. Thomas, Owen S. Gwinn, Megan L. Bartlett, Michael E. R. Nicholls

**Affiliations:** 1 Turner Institute for Brain and Mental Health, Monash University, Melbourne, AU; 2 College of Education, Psychology and Social Work, Flinders University, Adelaide, AU

**Keywords:** Attention, Spatial cognition, Social cognition

## Abstract

Pseudoneglect causes neurologically intact individuals to bias their attention to the left in near space, and to the right in far space. These attentional asymmetries impact both ambulatory and non-ambulatory activities, causing individuals to deviate rightward. While most studies investigating real-world navigation have found a rightward deviation when passing through a door, some have found the opposite pattern for corridors. To explore this dissociation, the current experiment explicitly compared navigation through doorways and corridors. To allow for a direct comparison between these two environments, the navigation task was undertaken in a simulated environment. Dextral participants (*n* = 98) completed several trials in either the doorway or corridor condition and their mean lateral position and variance was analysed. A rightward deviation was observed for doorways, consistent with previous research. Rightward biases were also observed for corridors, irrespective of the position within the corridor. The results argue against an explanation based on near/far space for the leftward bias in corridors. An explanation based on elevation of view is proposed as an alternative. The study also demonstrates that simulated environments provide an efficient means of investigating asymmetries in navigation.

## 1. Introduction

It is well established that our visuospatial attention is not directed in a perfectly symmetrical fashion. Indeed, most neurologically normal individuals show a small, but reliable, attentional bias toward the left side of near space on various tasks of visuospatial attention (McCourt, 2001). This attentional bias is known as pseudoneglect (Bowers & Heilman, 1980; Jewell & McCourt, 2000; Learmonth, Gallagher, Gibson, Thut, & Harvey, 2015). Although these asymmetries were initially recorded on pencil-and-paper and computerised tasks in the laboratory, more recent research has demonstrated that these asymmetries also extend to real-world behaviours, such as sporting performance (see Churches & Nicholls, 2016 for a review), driving (Friedrich, Elias, & Hunter, 2017; Learmonth, Märker, McBride, Pellinen, & Harvey, 2018), and navigation (Nicholls, Loftus, Mayer, & Mattingley, 2007; Nicholls et al., 2010; Robertson, Forte, & Nicholls, 2015; Thomas et al., 2017).

There has been much debate surrounding the neural mechanisms that underlie attentional asymmetries, with a primary focus on the role of the right hemisphere in visuospatial attention (Corbetta & Shulman, 2011; Kinsbourne, 1970; Thiebaut de Schotten et al., 2011; Zago et al., 2017). Current theories are supported by neuroimaging evidence, which shows that the right hemisphere is indeed more active during visuospatial attention tasks (Çiçek, Deouell, & Knight, 2009; Fink et al., 2000; Fink, Marshall, Weiss, & Zilles, 2001; Foxe, McCourt, & Javitt, 2003; Zago et al., 2017). Furthermore, individuals with stronger parieto-frontal connections in the right hemisphere (as compared to the left) produce more leftward line bisection errors (Thiebaut de Schotten et al., 2011) and the degree of leftward asymmetry in line bisection is positively correlated with the amount of right hemisphere activation (Zago et al., 2017). Collectively, these findings provide strong support for the role of the right hemisphere in visuospatial attention and pseudoneglect.

Nicholls et al. (2007) were the first to examine whether participants display asymmetries in collision behaviour during navigation in an experimental setting. They constructed a free-standing doorway and asked participants to pass through the doorway while shooting a toy-gun. When participants squeezed the trigger on the toy-gun bimanually, more rightward collisions were reported. In a follow-up experiment (Nicholls, Loftus, Orr, & Barre, 2008), participants entered digits into a cordless phone while walking through the doorway. Once again, Nicholls et al. (2008) found participants were more likely to collide on the right side as compared to the left.

To explain these navigation asymmetries, Nicholls et al. (2010) suggested that the relatively more rightward biases that are observed when line bisection is performed in far space (Longo & Lourenco, 2006; McCourt & Garlinghouse, 2000) could explain the occurrence of the rightward collision bias. If participants mentally bisect the doorway prior to commencing navigation and this bisection is right of centre, participants might then veer rightward (i.e., toward their bisection point) and ultimately collide with the doorway on the right during passage. To investigate this possibility, Nicholls et al. (2010) developed a novel paradigm wherein participants navigated a wheelchair or an electric scooter through a doorway. Across six experiments, consistent rightward biases were observed, leading the authors to conclude that a rightward bisection of the doorway in far space provided a good explanation of their findings. This explanation is further strengthened by research showing that participants look to the right when navigating an aperture and mark the centre to the right of true centre on these trials (Robertson et al., 2015).

Rightward deviations during navigation have been replicated in other laboratories. In 2011, Fujikake, Higuchi, Imanaka, and Maloney conducted three experiments to better understand the role of motor biases in collision asymmetries. Their findings indicated that rightward collisions occurred regardless of leading foot, although the bias toward the right was stronger when the right foot passed through the doorway first. The remainder of their results showed that unimanual activity shifted collisions contralaterally, which was consistent with the unimanual data reported by Nicholls et al. (2007, 2008).

Some researchers, however, have failed to observe a rightward navigation asymmetry. Hatin, Tottenham, and Oriet (2012) examined collision biases using a real-world navigation task that was modelled after Nicholls et al. (2008). To test whether the bias was related to pseudoneglect, Hatin et al. (2012) manipulated age, cueing and motor-activity, all of which are known to affect the magnitude of pseudoneglect. Of these manipulations, only motor activity attenuated the navigation asymmetry. More importantly, however, they unexpectedly observed a leftward deviation in both of their experiments. Hatin et al. (2012) suggested that this leftward deviation was related to the environment in which the study was carried out.

While the studies by Nicholls et al. (2007, 2008, 2010) placed the doorway in the middle of an empty room, Hatin et al. (2012) placed the doorway within a corridor. They suggested that this arrangement could have two effects. First, the arrangement of a doorway within a corridor could encourage participants to elevate the focus of their attention away from the floor towards the sides of the space. There is a connection between left/upper and far space and a connection between right/lower and near space (Nicholls, Mattingley, Berberovic, Smith, & Bradshaw, 2004; Thomas & Elias, 2010) possibly mediated by the asymmetrical neurological layout of the ventral and dorsal visual streams (Nicholls, Beckman, & Churches, 2016; Thomas, Schneider, Gutwin, & Elias, 2012). Second, Hatin et al. (2012) suggested that the corridor could affect the coding of near and far space, which is thought to be critical to the rightward navigation bias (Nicholls et al., 2010). In the studies by Nicholls et al. (2007, 2008, 2010) participants started walking towards the doorway in open space, which may have encouraged coding of the doorway as being in far space – producing a rightward bias. In contrast, the study by Hatin et al. (2012) required participants to start walking towards the doorway while standing within a corridor. The confined space of the corridor may have encouraged participants to code all the space as being in near space – leading to a leftward bisection bias (see Nicholls et al., 2016).

The leftward deviation observed by Hatin et al. (2012) is an important exception to the rightward deviation that has generally been observed for aperture navigation. To explore why this dissociation occurred, the current experiment explicitly compared the effect of bisecting a corridor compared to a door. While previous research in this area has made use of an actual doorway, which is arguably ecologically valid, we can also see that the mixed findings in previous research suggest that methodological differences in the physical set-up influence performance. Furthermore, the use of an actual doorway is cumbersome to construct and requires a significant amount of space. Therefore, we used a computerised point-of-view maze task, where the free-standing doorway and a corridor could be compared directly and easily. Research by Gamberini, Seraglia, and Priftis (2008) demonstrates that similar outcomes can be obtained using both real-world and virtual environments. They asked participants to bisect lines in real and simulated environments and found similar leftward/rightward biases in near/far space – irrespective of the environment in which the lines were presented. Although we have not used an immersive 3D environment herein, prior research suggests that computer-based testing can be used to simulate real-world experiences (e.g., Moffatt, Zonderman, & Resnick, 2001; Péruch & Gaunet, 1998; Thomas et al., 2009). Indeed, Ruddle, Payne, and Jones (1997) showed that simulated environments provide a reliable measure of real-world behaviour (see Maguire, Burgess, & O’Keefe, 1999 for a review).

In order to examine how, and also why, performance differs between the doorway and corridor conditions, we examined bisections in the very middle of the doorway and corridor (i.e., *midpoint location*) as well as where the doorway and corridor started (i.e., *early location*). The early location data indicate the location along the x axis as participants enter the corridor in the corridor condition and before they enter the doorway in the doorway condition. The early location in the doorway condition was equivalent to the beginning of the corridor in the corridor condition. If the coding of near and far space affect deviations between doors and corridors, a dissociation between the midpoint and early location data should be observed. For the *doorway* condition, no deviation would be predicted in the early location data because the doorway is still some distance away. A rightward deviation was expected, however, for the midpoint location as participants crossed through the doorway. A different pattern of results was predicted for the *corridor* condition. For the early location data, a rightward deviation can be predicted as participants code the aperture in far space as they head towards it and mis-bisect the aperture to the right. When inside the corridor, however, a leftward bias can be predicted for the midpoint location because space is coded as being ‘near’. To increase task variability and reduce fatigue, participants navigated through doorways and corridors of three different widths. In line with the pseudoneglect literature (Jewell & McCourt, 2000) and research on navigation (Nicholls et al., 2010), an increase in the deviation was expected for wider gap widths.

In addition to analysing the mean bisection point, the variation of bisections was also assessed using the standard deviation (*SD*) of responses. This measure provides an indication of error/variability in each of the conditions and provides a validation of the methodology. For the doorway condition, it was expected that the *SD* would be high in the early location data before participants entered the doorway, but would be reduced as participants entered the doorway at the midpoint. For the corridor condition, it was expected that there would be no difference in *SD* between the early and midpoint locations. It was also expected that *SD* would increase as a function of increases in gap width.

Finally, the association between deviations in the different conditions were assessed. Although the correlation between different measures of pseudoneglect is disappointingly low (Heber, Siebertz, Wolter, Kuhlen, & Fimm, 2010; Learmonth et al., 2015; Luh, 1995; Nicholls, Bradshaw, & Mattingley, 1999), Nicholls, Jones, and Robertson (2016) observed an association between the deviation for a remote-controlled vehicle and bisection of lines in far space. More recently, Learmonth et al. (2018) found a moderate negative correlation between performance on the landmark task and a computer-based lane keeping task. With this in mind, a near-space line bisection task was incorporated in the protocol. It was anticipated that there would be a leftward bias for the line bisection task and that this would be negatively correlated with the doorway and corridor conditions where a deviation was expected. We also tested the correlation between the early and the midpoint location data in the doorway and corridor conditions to determine whether participants were consistent within the navigation tasks.

## Method

### 2.1. Participants

A total of 108 undergraduate psychology students at Flinders University, with a mean age of 26.96 years (*SD* = 10.88), took part in the study. All were right-handed as determined by the Flinders Handedness Survey (FLANDERS; Nicholls, Thomas, Loetscher & Grimshaw, 2013). Optical corrections were worn if required. Data were missing for 10 participants due to hardware issues. These participants were removed from all further analyses, resulting in *n* = 98 (21 males). This left 45 participants in the *doorway* condition and 53 participants in the *corridor* condition.

### 2.2. Apparatus and Stimuli

#### 2.2.1. Threshold bisection task

A computer-based virtual room was constructed using MazeSuite 3.0.1 (Ayaz, Allen, Platek, & Onaral, 2008). This room was 20 × 20 units, with one virtual maze unit approximating 1.5 m in the real-world. The midline of the room was marked by a white line on the floor at 10 units along the z-axis and divided by a wall, which contained a central gap of three possible widths: 2 (narrow), 2.5 (medium), and 3 (wide) units. In a first *doorway* condition the dividing wall had a thickness of 0 units. In a second *corridor* condition the dividing wall had a thickness of 8 units. The starting position for each trial was the back wall, at 0 units along the z-axis. Exact starting position on each trial varied, such that the participant could begin either 1.5 units to the left or right of the midline of the room along the x-axis. The visual angle of the central gap necessarily changed as participants navigated the space during the task. However, from the starting position on the back wall, the three gap widths at the point of the white line subtended approximately 7.5°, 9.3°, and 11.1° visual angle.

**Figure 1:**
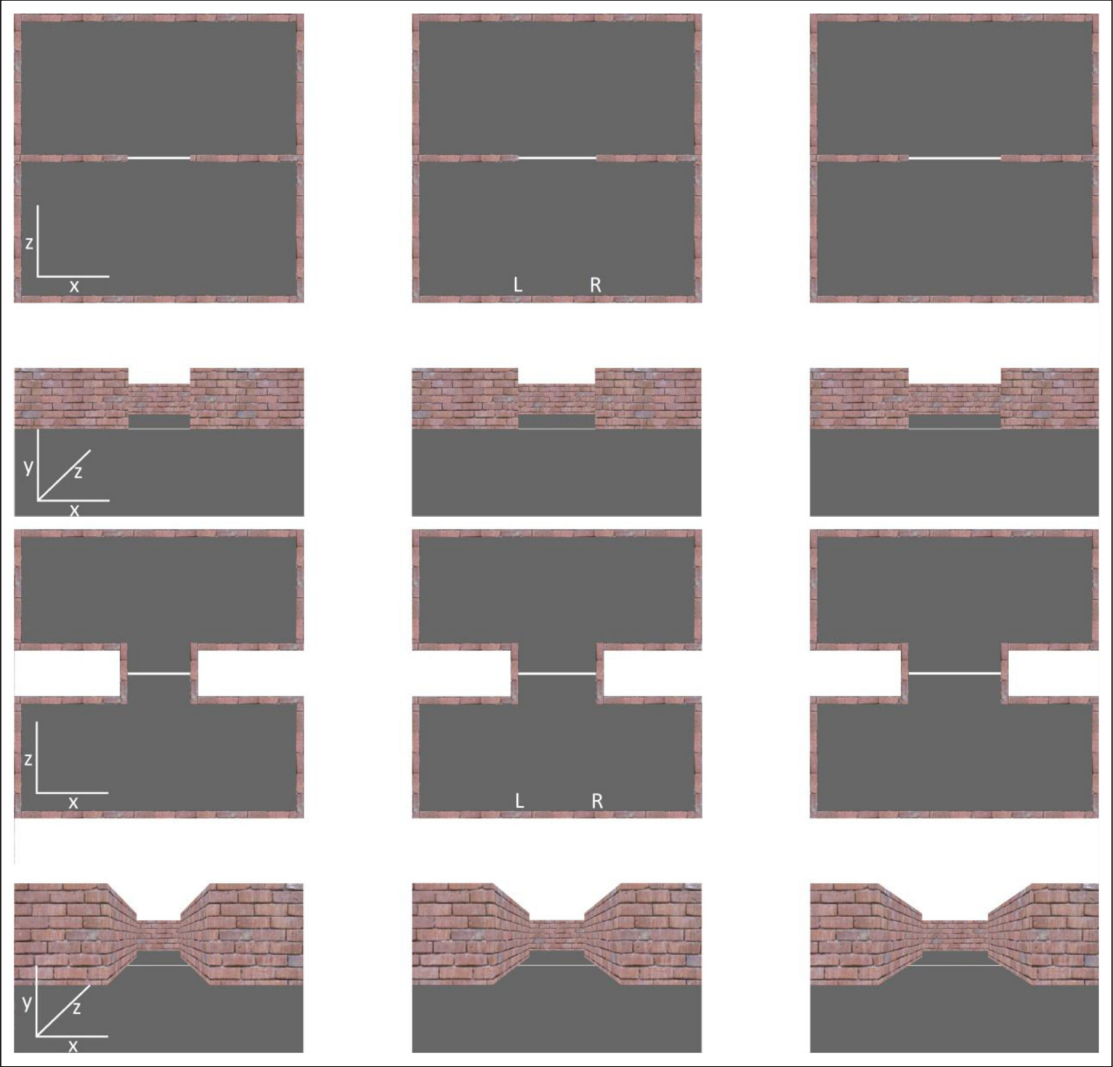
Sample top-down and first-person views of the room for all three gap widths for the *doorway* (upper panel) and *corridor* (lower panel) conditions. The upper row in each panel shows a top-down view of the layout of the room. The lower row in each panel shows the first-person view of the room when positioned along the exact midline of the room (note that in the actual experiment starting positions were always to the left or right of centre, as indicate by the labels ‘L’ and ‘R’). Columns in each panel show the three gap widths. Axis labels ‘x, y, z’ indicate the realtive orientation within each view.

**Figure 2:**
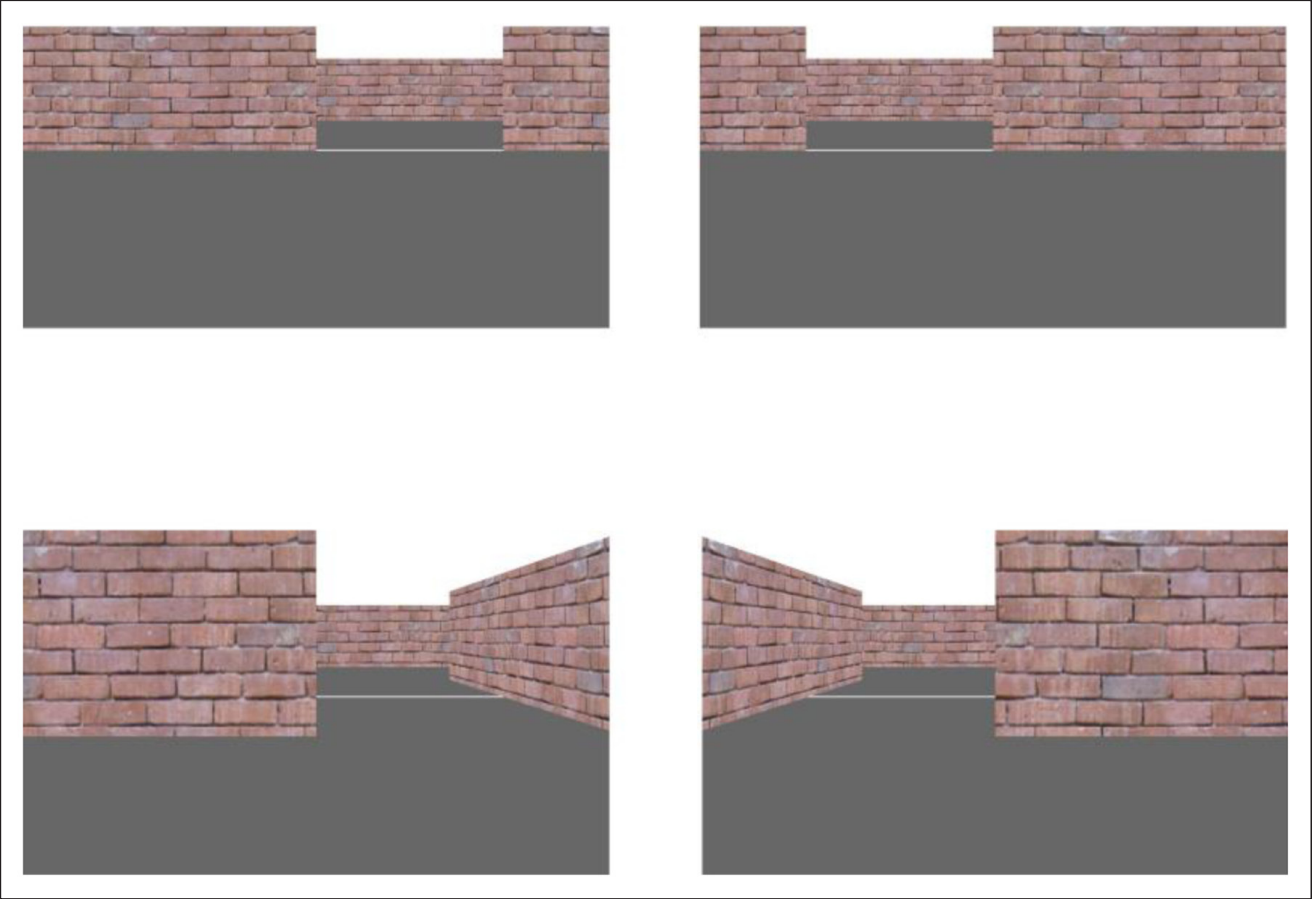
Sample first-person views of the wide gap width from the left (leftmost panel) and right (rightmost panel) starting positions for the *doorway* (upper panel) and *corridor* (lower panel) conditions.

Participants navigated the room using a Logitech F310 Gamepad (Logitech, Switzerland). Forwards/backwards movements along the z-axis were controlled using the left joystick, while left/right viewpoint rotations around the y-axis were controlled using the right joystick. Vertical viewpoint changes around the x-axis and left/right strafing movements along the x-axis were disabled. Figure 1 shows sample top-down and first-person views of the room for all three gap widths for the *doorway* (upper panel) and *corridor* (lower panel) conditions. Figure 2 shows sample first-person views of the wide gap width from the left (leftmost panel) and right (rightmost panel) starting positions for the *doorway* (upper panel) and *corridor* (lower panel) conditions.

#### 2.2.2. Line bisection task

Ten white lines, of three different lengths [3 lines measuring 600 (6.6° visual angle), 3 measuring 700 (7.7°), and 4 measuring 800 (8.8°) pixels] were generated on black backgrounds using Matlab 2013b (MathWorks, USA).

### 2.3. Procedure

Testing sessions were conducted in groups of 3–10 participants. All stimuli were presented on Dell LCD monitors (Dell, USA) with a resolution of 1680 × 1050 pixels, and were viewed at a distance of approximately 570 mm. Each testing station was flanked by dividers such that participants could not see the monitors to either side of them. Following informed consent, participants completed a demographic questionnaire and the FLANDERS survey; participants were then told they would see lines of varying length and would be asked to indicate the centre of each line via a mouse click, using the index finger of the right hand. Although response hand can influence perceived line midpoint (McCourt, Freeman, Tahmahkera-Stevens, & Chaussee, 2001), we chose to maintain the standard mouse mapping commonly used by right-handers. There was no time limit to respond and participants could change their response as many times as they wished before clicking a ‘confirm’ button to proceed to the next trial. The cursor then re-appeared in the same location as the previous ‘confirm’ button, and this button appeared with equal frequency on the left and right side of the screen. The location of each line was jittered along the x-axis of the monitor across trials; the y-axis position was held at 50% of the monitor’s height. This procedure ensured the centre of each line was not always in the same location.

Following the line bisection task, participants completed the threshold bisection task. The doorway and corridor conditions were run between-participants. All participants were asked to navigate through a gap in a wall with a white line on the floor, while attempting to cross the white line as close to its centre as possible. The block began with six practice trials, comprising one trial of each of the three gap widths, at both left and right starting positions, with no feedback. Following the practice trials, participants completed 60 experimental trials, comprising 20 trials (10 left start, 10 right start) for each of the three gaps. Each trial terminated .25 units after passing through the doorway or exiting the corridor. Each participant saw the same order of randomised trials to ensure any potential practice effects were similar across participants. Participants were debriefed after the testing sessions, which lasted between 15 and 30 minutes.

## 3. Results

Deidentified data are available on the Open Science Framework, https://osf.io/by39h/.

### 3.1. Response asymmetries

For the line bisection task, the midpoint of each line was defined as a zero-point origin from which participants’ bisections could form a negative (leftward bias) or positive (rightward bias) deviation. These deviations were recorded as a percentage of the total line length and were averaged across line lengths to create a single bisection score for each participant. Deviations were averaged across line length as there were too few data points to analyse line length separately (*n* = 10 lines in total). Data were first submitted to a one-sample *t*-test to compare mean line bisection biases to zero. Analysis revealed a significant leftward bias (*M* = –.29, *SD* = 1.26), *t*(97) = 2.32, *p* = .023, *d* = .234.

Data for the threshold bisection task were also recorded as negative (leftward bias) or positive (rightward bias) percentage deviations from the midpoint of the door or corridor. These data were averaged across the three gap widths and two starting points for each participant, keeping the doorway and corridor conditions separate. Data from the doorway condition were submitted to two one-sample *t*-tests to compare mean threshold bisection bias at the early and midpoint locations to zero. Analysis revealed a non-significant leftward bias at the early (*M* = –.07, *SD* = .44), *t*(44) = 1.13, *p* = .263, *d* = .169, contrasting a significant rightward bias at the midpoint location (*M* = .13, *SD* = .40), *t*(44) = 2.17, *p* = .036, *d* = .323. Data from the corridor condition were then submitted to two one-sample *t*-tests to compare mean threshold bisection bias at the early and midpoint locations to zero. Analysis revealed significant rightward biases at both the early (*M* = .15, *SD* = .44), *t*(52) = 2.45, *p* = .018, *d* = .337, and midpoint (*M* = .20, *SD* = .39) locations, *t*(52) = 3.83, *p* < .001, *d* = .526.

### 3.2. Correlations

To further investigate the associations between mean threshold bisection biases and line bisection biases, data were submitted to a series of correlations. Table 1 presents Pearson correlations among mean threshold bisection and line bisection biases. Analysis revealed significant positive associations between mean early and midpoint location threshold bisection biases for both the doorway and corridor conditions. All other associations were non-significant.

**Table 1:** Pearson Correlations Among Mean Threshold Bisection and Line Bisection Biases.

Measure	1	2	3	4	5

1. Doorway Early point	_				
2. Doorway Midpoint	.749**	_			
3. Corridor Early point	_	_	_		
4. Corridor Midpoint	_	_	.799**	_	
5. Line Bisection	.064	–.009	.028	.106	_

** Correlation is significant at the 0.01 level (2-tailed).

### 3.3. Mean threshold bisection bias

Data from the threshold task were submitted to a 2 (location: early, mid) × 2 (starting position: left, right) × 3 (gap width: narrow, medium, wide) repeated-measures ANOVA to investigate the effects of location, starting position, and gap width on mean threshold bisection bias. Two separate ANOVAs were conducted for the *doorway* and *corridor* conditions.

**Figure 3:**
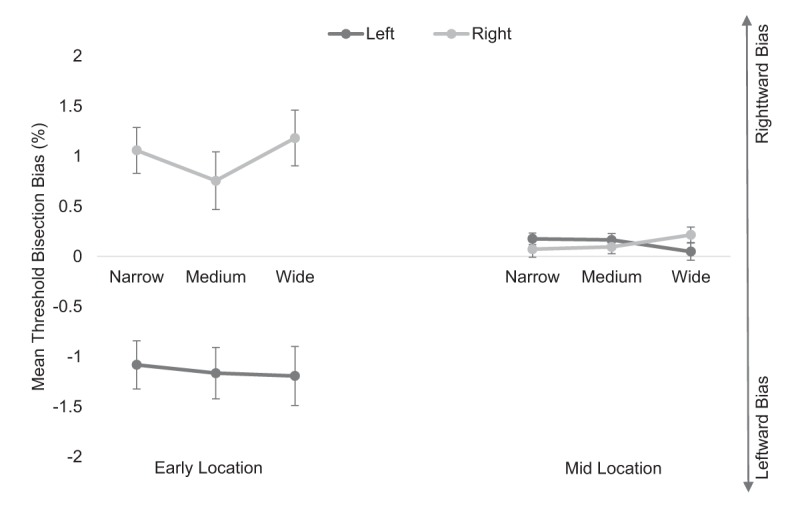
Mean threshold bisection bias (%) for the *doorway* condition at the early point (leftmost panel) and midpoint (rightmost panel) locations for the left (dark lines) and right (light lines) starting positions for each gap width. Error bars show standard errors.

Analysis for the *doorway* condition revealed a main effect of location, *F*(1, 44) = 20.67, *p* < .001, \eta _p^2 = .320 (see Figure 3). The main effect of starting position was also significant, *F*(1, 44) = 18.09, *p* < .001, \eta _p^2 = .291, whereby mean deviation was more leftward (*M* = –.51, *SE* = .14) when starting the task offset to the left and more rightward (*M* = .56, *SE* = .14) when offset to the right. The main effect of gap width was not significant, *F*(2, 88) = 1.79, *p* = .173, \eta _p^2 = .039.

The ANOVA also revealed a significant location by starting position interaction, *F*(1, 44) = 20.35, *p* < .001, \eta _p^2 = .316 (see Figure 3). To explore this interaction further, two separate one-way ANOVAs were conducted. For the early location data, analysis revealed a main effect of starting position, *F*(1, 44) = 19.45, *p* < .001, \eta _p^2 = .307, whereby mean deviation was more leftward (*M* = –1.15, *SE* = .25) when starting the task offset to the left and more rightward (*M* = 1.00, *SE* = .25) when offset to the right. For the mid location data, the main effect of starting position failed to reach significance, *F*(1, 44) = .002, *p* = .967, \eta _p^2 = .000.

The starting position by gap width interaction was also significant, *F*(2, 88) = 4.25, *p* = .017, \eta _p^2 = .088 (see Figure 3). To explore this interaction further, two separate one-way ANOVAs were conducted. For the right starting position, analysis revealed a main effect of gap width, *F*(2, 88) = 4.78, *p* = .011, \eta _p^2 = .098. Post hoc comparisons using a Bonferroni adjusted alpha level of .017 indicated that bias for the wide gap width was significantly more rightward than bias for the medium gap width (*p* = .002). The other two gap width comparisons were not significant (*p*’s > .464). For the left starting position, the main effect of gap width was non-significant, *F*(2, 88) = 1.07, *p* = .347, \eta _p^2 = .024. All other interactions were not significant (*p*s > .096).

**Figure 4:**
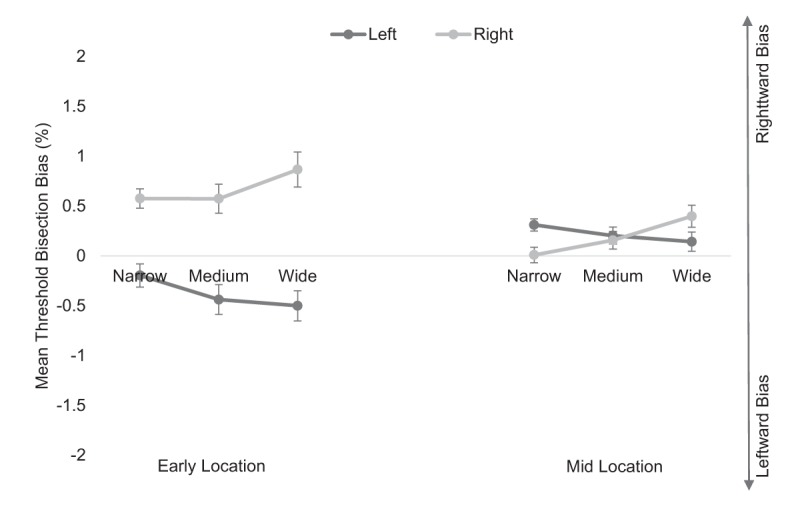
Mean threshold bisection bias (%) for the *corridor* condition at the early (leftmost panel) and midpoint (rightmost panel) locations for the left (dark lines) and right (light lines) starting positions for each gap width. Error bars show standard errors.

Analysis for the *corridor* condition revealed a main effect of starting position, *F*(1, 52) = 12.26, *p* = .001, \eta _p^2 = .191, whereby mean deviation was more leftward (*M* = –.08, *SE* = .09) when starting the task offset to the left and more rightward (*M* = .43, *SE* = .09) when offset to the right (see Figure 4). The main effects of location, *F*(1, 52) = 2.44, *p* = .125, \eta _p^2 = .045, and gap width, *F*(2, 104) = 2.78, *p* = .067, \eta _p^2 = .051, were non-significant. The location by starting position by gap width interaction was also non-significant, *F*(2, 104) = .14, *p* = .868, \eta _p^2 = .003.

The ANOVA also revealed a significant location by starting position interaction, *F*(1, 52) = 22.79, *p* < .001, \eta _p^2 = .305 (see Figure 4). To explore this interaction further, two separate one-way ANOVAs were conducted. For the early location data, analysis revealed a main effect of starting position, *F*(1, 52) = 20.23, *p* < .001, \eta _p^2 = .280, whereby mean deviation was more leftward (*M* = –.38, *SE* = .13) when starting the task offset to the left and more rightward (*M* = .67, *SE* = .13) when offset to the right. For the mid location data, the main effect of starting position failed to reach statistical significance, *F*(1, 52) = .072, *p* = .790, \eta _p^2 = .001.

The starting position by gap width interaction was also significant, *F*(1.64, 85.14) = 12.76, *p* < .001, \eta _p^2 = .197 (see Figure 4). To explore this interaction further, two separate one-way ANOVAs were conducted. For the right starting position, bisection increased linearly as a function of gap width, *F*(1, 52) = 11.74, *p* = .001, \eta _p^2 = .184. For the left starting position, bisection decreased linearly as a function of gap width, *F*(1, 52) = 13.43, *p* = .001, \eta _p^2 = .205.

The location by gap width interaction was also significant, *F*(2, 104) = 3.37, *p* = .038, \eta _p^2 = .061 (see Figure 4). To explore this interaction further, two separate one-way ANOVAs were conducted. Although the effect of gap width was not significant for the early location data, *F*(1, 52) = .01, *p* = .920, \eta _p^2 = .000, bisection increased linearly as a function of gap width at the midpoint location, *F*(1, 52) = 4.61, *p* = .037, \eta _p^2 = .081.

### 3.4. Mean threshold bisection variance

To obtain a measure of variance/error, the mean standard deviation for each participant in each of the conditions was calculated and analysed. Data from the threshold task were submitted to a 2 × 2 × 3 repeated-measures ANOVA to investigate the effects of location, starting position, and gap width on the variance on mean threshold bisection bias. As above, two separate ANOVAs were conducted for the *doorway* and *corridor* conditions.

**Figure 5:**
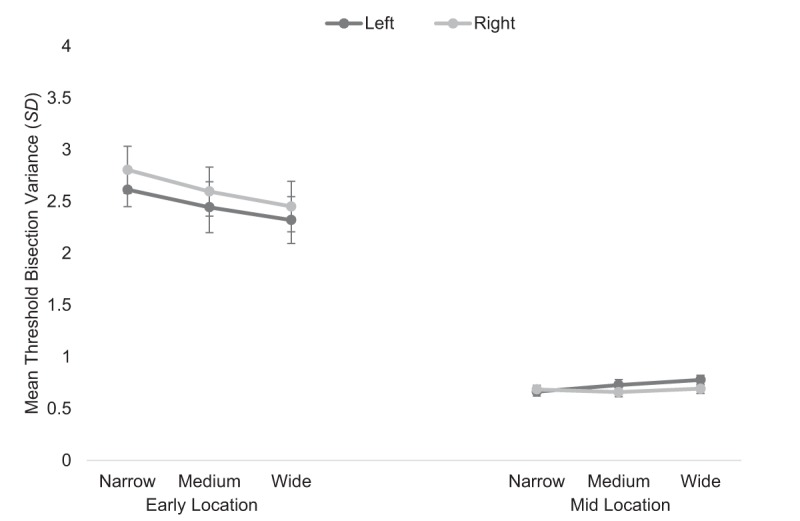
Mean threshold bisection variance (*SD*) for the *doorway* condition at the early (leftmost panel) and midpoint (rightmost panel) locations for the left (dark lines) and right (light lines) starting positions for each gap width. Error bars show standard errors.

Analysis for the *doorway* condition revealed a main effect of location, *F*(1, 44) = 82.93, *p* < .001, \eta _p^2 = .653 (see Figure 5), whereby the variance on mean bisection bias decreased from the early (*M* = 2.54, *SE* = .22) to the midpoint (*M* = .70, *SE* = .04). Analysis also revealed a main effect of gap width, *F*(2, 88) = 4.80, *p* = .010, \eta _p^2 = .098. To explore the main effect of gap width further, post hoc comparisons using Bonferroni adjusted alpha levels of .017 indicated that the variance for the narrow gap width was significantly greater than the variance for the wide gap width (*p* = .006). The other two gap width comparisons were not significant (*p*’s > .256). Lastly, the main effect of starting position was not significant, *F*(1, 44) = 3.43, *p* = .071, \eta _p^2 = .072.

The ANOVA also revealed a significant location by starting position interaction, *F*(1, 44) = 15.16, *p* < .001, \eta _p^2 = .256 (see Figure 5). To explore this interaction further, two separate one-way ANOVAs were conducted. For the early location data, analysis revealed a main effect of starting position, *F*(1, 44) = 9.01, *p* = .004, \eta _p^2 = .170, whereby the variance on bisection increased from the left (*M* = 2.46, *SE* = .23) to the right starting position (*M* = 2.62, *SE* = .20). For the mid location data, the main effect of starting position just reached statistical significance, *F*(1, 44) = 4.10, *p* = .049, \eta _p^2 = .085, whereby the variance on bisection decreased from the left (*M* = .72, *SE* = .04) to the right starting position (*M* = .68, *SE* = .04).

The location by gap width interaction was also significant, *F*(2, 88) = 9.73, *p* < .001, \eta _p^2 = .181 (see Figure 5). To explore this interaction further, two separate one-way ANOVAs were conducted. For the early location data, the variance on bisection decreased linearly as a function of gap width, *F*(1, 44) = 15.80, *p* < .001, \eta _p^2 = .264. For the mid location data, variance increased linearly as a function of gap width, *F*(1, 44) = 4.40, *p* = .042, \eta _p^2 = .091. All other interactions were not significant (*p*’s > .617).

**Figure 6:**
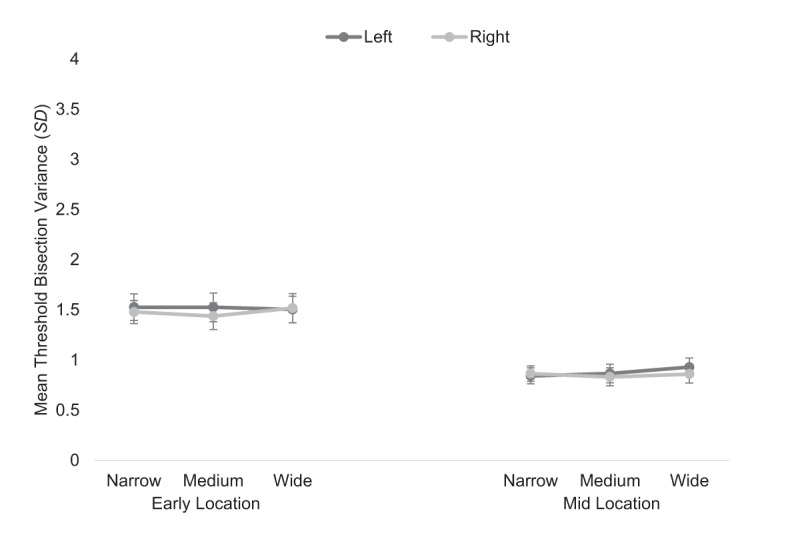
Mean threshold bisection variance (*SD*) for the *corridor* condition at the early (leftmost panel) and midpoint (rightmost panel) locations for the left (dark lines) and right (light lines) starting positions for each gap width. Error bars show standard errors.

Analysis for the *corridor* condition revealed a main effect of location, *F*(1, 52) = 43.17, *p* < .001, \eta _p^2 = .454 (see Figure 6), whereby the variance on mean bisection bias decreased from the early (*M* = 1.50, *SE* = .13) to the midpoint (*M* = .87, *SE* = .08). An assessment of Mauchly’s Test of Sphericity determined the parametric assumption of sphericity had been violated for the main effect of gap width, χ^2^(2) = 11.61, *p* = .003. To account for this inflation, a correction (ε = .83) was applied (Greenhouse & Geisser, 1959). The main effects of gap width, *F*(1.66, 86.41) = .48, *p* = .584, \eta _p^2 = .009, and starting position, *F*(1, 52) = 1.82, *p* = .183, \eta _p^2 = .034, were non-significant. All interactions were not significant (*p*’s > .254).

## 4. Discussion

The present experiment investigated conflicting results regarding navigation asymmetries. While Nicholls and colleagues have observed a consistent rightward deviation when passing through an aperture in a variety of conditions (Nicholls et al., 2007, 2008, 2010, 2016), Hatin et al. (2012) observed a leftward deviation. To explain the discrepancy, Hatin et al. (2012) suggested that the leftward bias was caused by the study being carried out in a corridor. Being in a corridor during the task could cause the aperture to be coded in near space—causing a leftward bisection and deviation. This experiment directly tested this proposition in a computer-based maze environment. To that end, participants were asked to navigate through the centre of either a doorway or a corridor of various widths.

In the *doorway* condition, measures of lateral position were taken as the participant approached the doorway (early location) and as they entered the doorway (midpoint location). For the early location data, analysis of starting position revealed a greater leftward deviation for the left starting position compared to a greater rightward deviation for the right starting position. The starting position data revealed that participants did not deviate to the left or right as they approached the doorway. These findings are unsurprising as they are consistent with the starting position and suggest that participants deviate towards the centre over time. Although the rightward bias was expected to increase for wider gap widths (Nicholls et al., 2010), we only observed an effect of gap width for right side starting positions, which was consistent with our expectation. When participants started on the right, rightward deviations were greater for wide as compared to medium gap widths. This finding could reflect the fact that participants showed a rightward deviation overall, which was stronger when starting on the right side and therefore was further accentuated by the wider gap width.

This condition was also associated with a high level of variation (as indexed by the *SD* of each individual’s responses). When participants started on the right side, they showed a higher variance at this early location than when starting on the left. However, once participants reached the midpoint the variance had decreased and there was greater variance for the left starting position compared to the right. The midpoint data revealed a rightward deviation as participants passed through the doorway with a much smaller level of variability. Interestingly, variance *decreased* as gap width increased at the early location, and then variance *increased* at the midpoint location as gap width increased. These findings suggest that variance changes the least for wider gap widths over the course of the trial and the most for the narrower gap widths.

The difference in size of the *SD*s across these two time points is expected; at the early point, participants had more free space to navigate and therefore the margin of error was larger than it was at the midpoint. The rightward deviation for passing through doorways confirms the rightward deviation that has been observed by Nicholls and colleagues (2007, 2008, 2010, 2016) in a wide range of conditions and corroborates research showing that attentional asymmetries extend to computer-based environments (Gamberini et al., 2008; Learmonth et al., 2018; Thomas et al., 2009).

In the *corridor* condition, early location and midpoint location threshold bisection biases were also analysed separately. The early point data revealed that participants deviated to the right as they approached the corridor. This rightward bias is consistent with the proposition that participants mis-bisect to the right in far space and then head towards that point. As in the doorway condition, deviations were more rightward for the right start point and more leftward for the left start point for the early location data. Analysis of the midpoint data revealed that the rightward deviation observed at the early point persisted as participants passed through the corridor with a smaller level of variability.

In relation to gap width, the early point data also showed a relatively high level of variance, which increased as a function of gap width. This pattern of variance demonstrates that responses were more variable as participants entered the corridor and that this variability increased as the width of the corridor increased. Furthermore, when participants started on the right, deviation became increasingly more rightward as gap width increased. When starting on the left, deviations became increasingly more leftward as gap width increased.

The rightward bias in the midpoint of the corridor is the opposite of what was predicted. One explanation offered by Hatin et al. (2012) for their leftward bias was that the corridor brought the aperture into near space – therefore creating a leftward bisection bias. In the current study, it was reasoned that entering a simulated corridor would also bring the aperture into near space – causing a leftward deviation. While it is possible that the corridor did not create the impression of near space, Gamberini et al. (2008) observed robust effects of simulated near and far space on attentional asymmetries. It therefore appears that the alternative explanation offered by Hatin et al. (2012) is more plausible. In this case, Hatin et al. (2012) suggested that the corridor caused an elevation of attention away from the floor. Because of the link between the upper and left hemispaces (Nicholls et al., 2004; Thomas & Elias, 2010), this link could cause mis-bisections and deviations towards the left. It is likely that the simulated environment used in the current study did not cause an elevation in the focus, and this lack of elevation may explain why a leftward bias was not found. To investigate the effect of elevation, it would be interesting to manipulate the point of view along the y axis to determine if elevation affects the direction of the deviation.

Although it is possible that use of the right hand to adjust lateral position while navigating through the doorway/corridor increased rightward biases, we do not believe that use of the right hand can account for our findings. Arguably, as movement along the y-axis was always controlled by the right hand, there could have been relatively more left hemisphere activation as a result. However, movement along the z-axis was concurrently performed by the left hand and any activational differences as a result of motor activity would have been slight. Furthermore, biases were not consistently rightward, which suggests that an underlying difference in hemispheric activation cannot account for the observed differences between the doorway and corridor conditions.

Consistent with expectations, we found a significant leftward bias for the line bisection task. Despite finding this asymmetry—and observing an asymmetry for the threshold bisection task—there was no correlation between the tasks. The lack of correlation between tasks is consistent with prior research (e.g., Heber et al., 2010; Learmonth et al., 2015; Luh, 1995; Nicholls et al., 1999), which has also failed to show a significant relationship between line bisection and real-world performance. One exception is the study by Nicholls et al. (2016) where a positive correlation was observed between rightward navigation asymmetries and right bisection biases in far space. The critical difference here may be that the line bisection was carried out in near space in the current study whereas it was carried out in far space in the study by Nicholls et al. (2016). It is known that processing of objects in near and far space engages different neural mechanisms (Weiss et al., 2000) and that these differences may obscure any correlation between the tasks. As expected, early and midpoint deviations did correlate significantly within the doorway and corridor conditions, suggesting that participants were consistent within the threshold bisection tasks.

Another exception was recently observed by Learmonth et al. (2018), who reported a moderate negative correlation between performance on the landmark task and a computer-based lane keeping task. Leftward asymmetries on the landmark task were associated with rightward biases in lane position amongst younger adults (18 to 34 years). Although this finding is rather interesting, both the lane keeping task and the landmark task differ from the tasks used herein. Importantly, Learmonth et al. failed to observe a significant correlation between lane keeping and line bisection.

An important limitation of our task was the use of a 2D computer-based maze task rather than a true immersive virtual environment. As a result, we cannot be certain that our participants perceived the environment in the same manner as they might have in the real-world. Namely, the doorways and corridors on the screen might not have been perceived as being representative of near and far space as the participants navigated forward. Although we cannot be certain of this perception, our findings provide preliminary evidence for the feasibility of using a computer-based task, which is more economical and practical than the use of virtual reality or real-world setups. Furthermore, previous research shows that performance in simulated environments relates to real-world performance reliably (Maguire et al., 1999; Ruddle et al., 1997).

## 5. Conclusions

The current study demonstrates that participants navigating through either a doorway or corridor on a computer-based maze task deviate to the right. Left/right judgments are crucial for navigation, where a slight deviation to the right when driving a car or walking across the road could increase the risk of a collision. To reduce the chances of these collisions, strategies should be developed in training or by the use of multisensory cues to re-orient attention (Ho & Spence, 2005; Klein & Shore, 2000; Spence & Driver, 2004). Furthermore, our findings demonstrate that research conducted in simulated environments, which investigate attentional and navigational asymmetries (Gamberini et al., 2008; Heber, Siebertz, Wolter, Kuhlen, & Fimm, 2008), not only translates to, but may also offer a methodologically sound alternative to research conducted in real-world environments (Loomis, Blascovich, & Beall, 1999; Riva, 2002; Tarr & Warren, 2002).

## Data Accessibility Statement

Deidentified data are available on the Open Science Framework, https://osf.io/by39h/.
